# Exercise suppresses apoptosis for alleviating Parkinson’s disease: effects on pathophysiological molecular pathways

**DOI:** 10.3389/fnagi.2026.1810397

**Published:** 2026-03-27

**Authors:** Junfei Chen, Xin Liu, Yue Wu, Chunlong Wang, Cong Wang

**Affiliations:** 1 Jiangsu Research Institute of Sports Science, Nanjing, China; 2 College of Physical Education, Yangzhou University, Yangzhou, China

**Keywords:** apoptosis, autophagy, exercise, mitochondrial dysfunction, neuroinflammation, Parkinson’s disease

## Abstract

Parkinson’s disease (PD) is characterized by the progressive degeneration of midbrain dopaminergic neurons, with apoptosis representing the predominant mechanism of neuronal cell death among the various forms of cell death implicated in PD. The lack of effective strategies to inhibit neuronal apoptosis remains a major challenge in PD management, particularly given the limitations associated with current pharmacological interventions. Exercise has gained increasing attention as a potentially effective approach to reduce PD symptoms and may alter disease progression by regulating apoptosis. However, the exact molecular pathways by which exercise provides neuroprotective benefits in PD remain incompletely understood. This narrative review synthesizes current evidence from animal models and human studies on the molecular mechanisms by which exercise alleviates neuronal apoptosis in PD. Following a comprehensive literature search of PubMed, Web of Science, and Scopus databases, we critically evaluate the evidence for exercise-mediated regulation of three key interconnected pathways: mitochondrial function (AMPK/Sirt1/PGC-1α signaling), neuroinflammation (TLR/MyD88/NF-κB signaling), and autophagy (CaMKII/Beclin1/p62 signaling). We discuss the translational limitations of current animal studies, identify gaps in the literature including the predominance of treadmill-based protocols and limited human evidence, and propose an integrative framework linking these pathways to coordinated neuroprotection. Understanding these molecular interactions will inform the development of optimized, personalized exercise interventions for PD management.

## Highlights

- Apoptosis is a primary pathogenic component that drives the course of PD and is the predominant mechanism of neuronal cell death.
- Exercise is increasingly acknowledged as a beneficial approach for alleviating symptoms of PD and potentially influencing the disease’s progression by regulating neuronal apoptosis.
- Exercise protects against excessive neuronal apoptosis in PD by modifying important pathogenic pathways, including mitochondrial function, neuroinflammation, and autophagy.

## 1 Introduction

Parkinson’s disease (PD) is a prevalent neurodegenerative disorder that predominantly afflicts the elderly globally (Xu L. et al., 2025; Gao et al., 2025; Rizig and Salama, 2026). Regrettably, due to the intricate nature of its pathology and the irreversible course it takes, PD is considered incurable. Patients diagnosed with PD typically have an average life expectancy of 7–15 years (Armstrong and Okun, 2020), highlighting the significant impact of this disease on individuals. As of 2015, PD afflicted approximately 6.2 million individuals and resulted in about 117,400 deaths globally (Vos et al., 2016), and according to estimates, the number of people over 50 with PD will double by 2030 (Li et al., 2022). The hallmark motor symptoms of PD, including muscle rigidity, resting tremors, bradykinesia, and postural instability, remarkably impair the quality of life of affected individuals (Poewe et al., 2017). Additionally, sensory, autonomic, and cognitive impairments constitute significant non-motor phenotypes of PD (Kwon et al., 2025; Schapira et al., 2017; Safarpour et al., 2026). These symptoms synergistically exacerbate the multidimensional challenges of the disease, presenting key difficulties in comprehensive clinical management (Schapira et al., 2017; Chen et al., 2024; Aarsland et al., 2021). Two fundamental characteristics of PD are the gradual death of dopaminergic (DA) neurons in the substantia nigra pars compacta (SNpc) and the development of Lewy bodies (LBs) in these neurons (Zarkali et al., 2025). The accumulation of *α*-synuclein (α-syn) in nerve cells disrupts their function, giving rise to pathological processes including mitochondrial dysfunction, neuroinflammation, autophagy inhibition, and protein dysregulation, which contribute to excessive loss of neurons, culminating in the manifestation of motor and non-motor symptoms observed in PD (Wood, 2019; Fang et al., 2025; Wood, 2025; Hallqvist et al., 2025; Lin et al., 2025; Lv et al., 2023; Haque et al., 2022; Bellini et al., 2025). Despite advancements in pharmacological treatments (Menozzi and Schapira, 2025), traditional medications can only offer partial relief from symptoms, with long-term usage potentially leading to drug resistance and various adverse effects like nausea and hallucinations (Armstrong and Okun, 2020; Chen et al., 2024; Fyfe, 2025).

Exercise has emerged as a promising non-pharmacological intervention for PD, with accumulating evidence demonstrating its neuroprotective effects and capacity to alleviate both motor and non-motor symptoms (Chen X. et al., 2025; Wilson et al., 2025; Diaz-Galvan et al., 2026). Clinical studies have consistently reported functional improvements in PD patients participating in aerobic exercise interventions (Zhen et al., 2022; Wei et al., 2025; Kim R. et al., 2025; Xie et al., 2025). Nicolien and colleagues reported that a 6-month aerobic exercise intervention had a significant functional improvement in sedentary PD patients aged 30–75 years (van der Kolk et al., 2019). The neurobiological basis of exercise intervention in PD remains under investigation. Currently, mounting evidence points to its role in mitigating excessive neuronal apoptosis, a key pathological event, suggesting this represents a crucial intervention target for exercise’s protective effects (Ferreira et al., 2021; Li N. et al., 2025; Wang B. et al., 2025). In PD, apoptosis-related factors (including Bax, Bcl-2, and Caspase-3) are frequently expressed abnormally (Erekat, 2018; Lei et al., 2025). The SNpc, corpus striatum (CS), hippocampus, cortex, and cerebrospinal fluid are among the important brain regions affected by this phenomena (Dionisio et al., 2021; Yin et al., 2025; Ishaq et al., 2024). Interestingly, interventions targeting anti-apoptotic pathways have shown promise in mitigating dopaminergic neuronal damage and reducing degeneration (Liu J. et al., 2020; Wang et al., 2024; Meng et al., 2024; Charvin et al., 2018).

Given the intricate regulation of apoptosis and its crucial role in exercise therapies, this paper aims to elucidate apoptosis as a core mechanism through which exercise improves PD pathology via a systematic review, thereby consolidating scientific consensus in this field. Therefore, the present study explored the molecular mechanisms of excessive neuronal apoptosis in PD ameliorated by exercise, mainly including mitochondrial dysfunction, neuroinflammation, and autophagy inhibition, which are closely related to it, aiming to provide an important target and theoretical research direction for the prevention and treatment of PD by exercise.

This article is a narrative review that synthesizes current knowledge on the molecular mechanisms by which exercise modulates neuronal apoptosis in PD. A comprehensive literature search was performed using PubMed, Web of Science, and Scopus databases for articles published up to March 2026. The search terms included combinations of the following keywords: “Parkinson’s disease,” “exercise,” “physical activity,” “treadmill training,” “apoptosis,” “programmed cell death,” “mitochondria,” “neuroinflammation,” and “autophagy.” Articles were included if they were original research or reviews published in English and investigated the relationship between exercise and apoptotic pathways in PD. Reference lists of relevant articles were also screened for additional studies. Given the narrative nature of this review, no formal quality assessment or meta-analysis was performed.

## 2 From PD pathogenesis to exercise intervention: the centrality of apoptosis

### 2.1 Pathogenic landscape of PD: where apoptosis fits

PD results from complex interactions between genetic susceptibility and environmental factors, culminating in progressive DA neuron loss. Over 20 genes have been implicated in PD pathogenesis, including SNCA, PINK1, DJ-1, PRKN, LRRK2, and ATP13A2 (Selvaraj and Piramanayagam, 2019; Kim et al., 2024; Fiume et al., 2025). These genes converge on cellular pathways essential for neuronal survival: mitochondrial quality control, protein homeostasis, and stress responses (Haque et al., 2022; Selvaraj and Piramanayagam, 2019; Ryan et al., 2015; Goyani et al., 2024). Importantly, each of these pathways ultimately connects to the regulation of apoptosis—the final common pathway of neuronal death in PD (Michel et al., 2016).

*α*-syn aggregation, the pathological hallmark of PD, exemplifies this convergence (Ghosh et al., 2017; Conway et al., 1998). Misfolded α-syn accumulates in neurons, forming Lewy bodies and, more importantly, presynaptic oligomers that disrupt synaptic function (Koga et al., 2021; Schmitz et al., 2023). This accumulation triggers multiple stressors: it impairs mitochondrial complex I activity, induces endoplasmic reticulum stress, activates microglial inflammatory responses, and overwhelms autophagic clearance mechanisms (Marchetti, 2020; Yi et al., 2022; Kuo et al., 2025; Mendonca and Peixoto, 2025). Each of these stressors can independently activate apoptotic cascades, positioning *α*-syn pathology as an upstream trigger of apoptosis rather than a direct executioner.

### 2.2 Apoptosis as the common pathway

Apoptosis in PD neurons proceeds through both extrinsic and intrinsic pathways, with the mitochondrial (intrinsic) pathway predominating (Ashkenazi, 2015; Lossi, 2022). Mitochondrial dysfunction—whether from genetic mutations (PINK1, PRKN), environmental toxins, or *α*-syn—reduces mitochondrial membrane potential (Δψm), increases outer membrane permeability, and releases cytochrome c into the cytoplasm (Spierings et al., 2005; Garrido et al., 2006; Tait and Green, 2010). Cytochrome c binds Apaf-1, forming the apoptosome that recruits and activates caspase-9, which in turn cleaves executioner caspase-3 (Xu et al., 2021). This cascade dismantles cellular structures, producing the characteristic morphological features of apoptosis: cell shrinkage, chromatin condensation, and DNA fragmentation (Mustafa et al., 2024).

Critically, apoptosis is not merely a passive consequence of cellular stress but an actively regulated process susceptible to modulation. The Bcl-2 protein family governs mitochondrial outer membrane permeability, with pro-apoptotic members (Bax, Bak, and Bad) promoting cytochrome c release and anti-apoptotic members (Bcl-2, Bcl-xl, and Mcl-1) inhibiting it (Hussein, 2005; Czabotar and Garcia-Saez, 2023; Green, 2022a). Caspase activity is similarly regulated, with inhibitor of apoptosis proteins (IAPs) providing endogenous inhibition (Yang and Li, 2000). This regulatory complexity creates opportunities for therapeutic intervention—including exercise—to tip the balance toward neuronal survival.

### 2.3 Evidence for excessive apoptosis in PD

Postmortem studies consistently demonstrate apoptotic markers in PD brains. Increased expression of pro-apoptotic proteins (Bax, caspase-3, caspase-8, and Par-4) and decreased anti-apoptotic factors (Bcl-2 and Bcl-xl) have been documented in substantia nigra, striatum, and hippocampus of PD patients (Erekat, 2018; Lei et al., 2025; Dionisio et al., 2021; Yin et al., 2025; Ishaq et al., 2024) (Table 1). TUNEL-positive neurons, indicative of DNA fragmentation, are observed in affected regions (Tatton et al., 2003; Tatton et al., 1998). These findings are recapitulated in animal models: rodents and primates exposed to MPTP, 6-OHDA, or rotenone exhibit upregulation of caspases, Bax, and cytochrome c release, alongside DA neuron loss (Table 1). Cell culture studies confirm that MPP^+^ (the active metabolite of MPTP) directly induces apoptosis in dopaminergic cell lines, with Bcl-2 overexpression conferring protection (Rani et al., 2023; Qi et al., 2021; O'Malley et al., 2003).

**Table 1:** Experimental evidence for the occurrence of excessive apoptosis in Parkinson’s disease.

Refs.	Type	PD model	Changes in apoptosis
Hartmann et al. (2000)	Human	postmortem human brain	Caspase-3 ↑
Hartmann et al. (2001)	Human	postmortem human brain	Caspase-8 ↑
Hartmann et al. (2002)	Human	postmortem human brain	Bcl-xl mRNA transcripts ↑
Tatton (2000)	Human	postmortem human brain	Bax, Caspase-3 ↑
Balakrishnan et al. (2021)	Animal	ROT induced Wistar rats	Cyt C ↑ Bax, Caspases-3, 8, 9↑
Javed et al. (2020)	Animal	ROT induced Wistar rats	Bax, cleaved Caspase-3, 9 ↑ Bcl-2 ↓
Wang et al. (2020)	Animal	MPTP^+^ induced C57BL/6 mice	Bax, Caspase-3, 8, 9 ↑ p-JNK and p-P38 ↑ Bcl-xl, Bcl-2 ↓
Duan et al. (1999)	Animal	MPTP^+^ induced adult rhesus monkeys	Par-4 ↑
Sun et al. (2022)	Cell	MPTP^+^ treated SH-SY5Y cells	Bax, cleaved Caspase-3 ↑ Bcl-2 ↓
Pouresmaeili-Babaki et al. (2018)	Cell	6-OHDA treated SH-SY5Y cells	Cyt C, cleaved Caspase-3 ↑ PI3K signaling pathway ↓
Peng et al. (2018)	Cell	MPTP^+^ treated SH-SY5Y cells	Tunel-positive cells ↑ Bcl-2/Bax ratio ↓
Zhang et al. (2019)	Cell	MPP^+^ treated PC12 cells	cleaved Caspase-3, PARP ↑ Annexin V-FITC positive cells ↑
Kong et al. (2024)	Cell	ROT treated SH-SY5Y cells	cleaved Caspase-3, 9, RARP ↑ Annexin V-FITC positive cells ↑
Zhou et al. (2021)	Cell	MPTP^+^ treated dopaminergic SK-N-SH neuroblastoma cells	Bax, cleaved Caspase-3 ↑ Bcl-2 ↓

↑, activation or increase; ↓, inactivation or decrease. Rot, rotenone; MPTP, 1-methyl-4-phenyl-1,2,3,6-tetrahydropyridine; 6-OHDA, 6-hydroxydopamine.

### 2.4 Why apoptosis is an attractive exercise target

Several features make apoptosis particularly suitable for exercise modulation. First, apoptosis is energy-dependent and regulated by cellular energy status (Shiraishi et al., 2001). Exercise, by enhancing mitochondrial biogenesis and cellular energetics (Vina et al., 2009; Irrcher et al., 2003), may directly influence the energetic threshold for apoptosis initiation. Second, apoptosis integrates signals from multiple stress pathways (Costa-Mattioli and Walter, 2020; Tabas and Ron, 2011). Exercise simultaneously targets mitochondrial function (Bishop et al., 2025; Grevendonk et al., 2021), neuroinflammation (Liu Y. et al., 2020; Liu et al., 2022), and autophagy (He et al., 2012)—the three major upstream regulators of apoptosis in PD—enabling coordinated suppression of apoptotic triggers. Third, unlike necrotic cell death, apoptosis preserves membrane integrity until late stages, allowing time for intervention (Kam and Ferch, 2000). This temporal window may permit exercise-induced adaptive responses to intervene before cell death becomes inevitable.

Thus, apoptosis represents not merely a downstream consequence of PD pathology but a central hub integrating diverse pathogenic signals. Its regulatory complexity and responsiveness to cellular physiology position it as a promising mechanistic target for exercise, which may exert neuroprotection by modulating the upstream pathways that drive apoptotic execution.

## 3 Regulatory mechanisms of apoptosis in PD

### 3.1 Mitochondrial dysfunction and apoptosis

**Figure 1:**
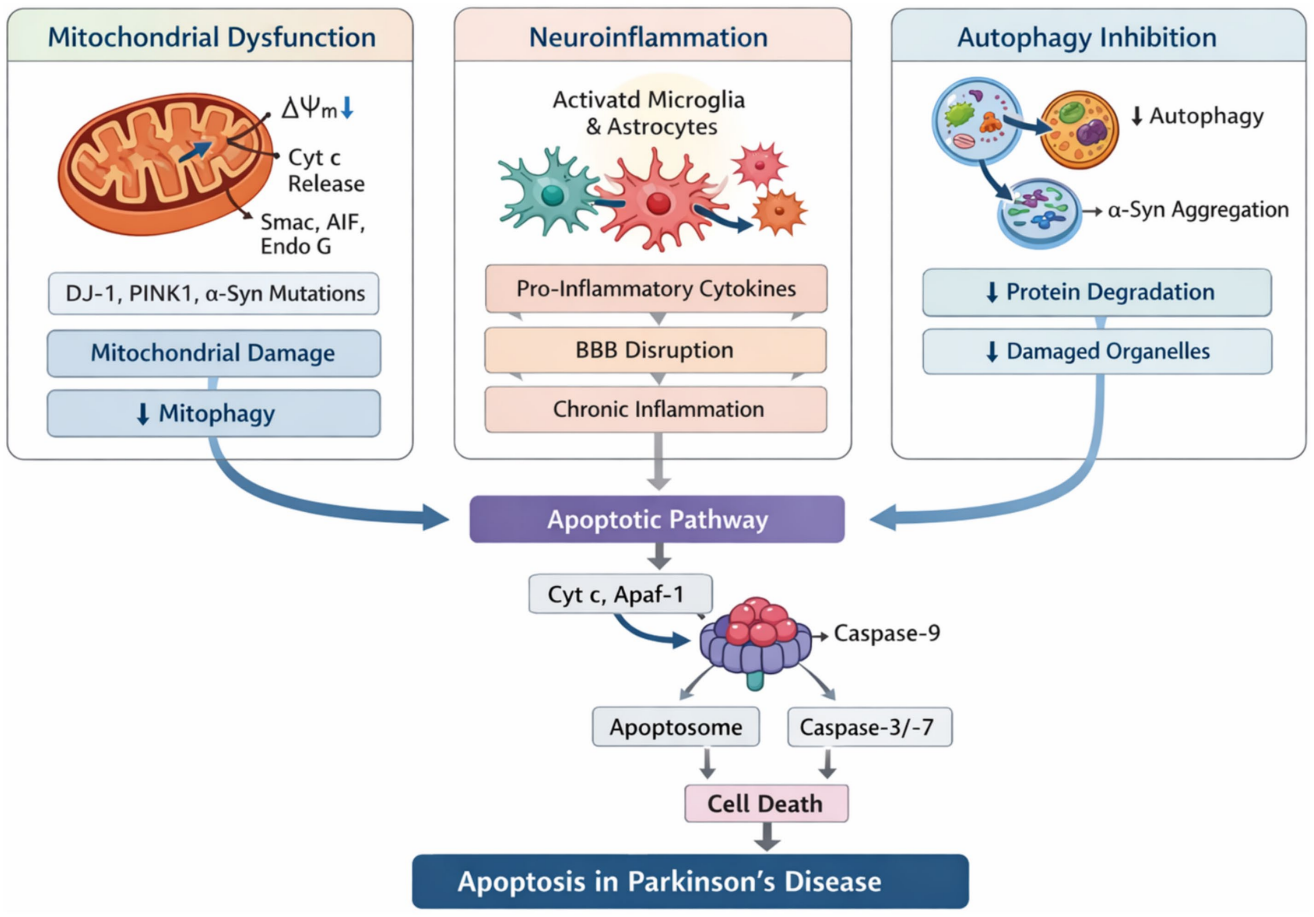
The main mechanism leading to excessive apoptosis of PD neurons. This diagram illustrates the three interconnected pathways leading to excessive neuronal apoptosis in PD and their convergence on the mitochondrial apoptotic cascade. Mitochondrial dysfunction pathway (left): PD-related insults (α-synuclein aggregation, genetic mutations) reduce mitochondrial membrane potential (Δψm) and increase outer membrane permeabilization, releasing cytochrome *c* (Cyt *C*) into the cytoplasm. Cyt *C* binds Apaf-1 to form the apoptosome, activating caspase-9 and downstream caspase-3. Pro-apoptotic Bcl-2 family members (Bax and Bak) promote this process, while anti-apoptotic members (Bcl-2 and Bcl-xl) inhibit it. Neuroinflammation pathway (middle): Aggregated α-synuclein activates microglia via Toll-like receptors (TLR2/4), triggering MyD88-dependent NF-κB activation. This promotes transcription of pro-inflammatory cytokines (TNF-α, IL-1β, and IL-6), which activate neuronal death receptors and caspase-8, converging on caspase-3. Autophagy-lysosome pathway (right): Impaired autophagy (reduced Beclin1, decreased LC3-II conversion) leads to accumulation of damaged mitochondria and toxic protein aggregates, indirectly promoting apoptosis. Caspase-mediated cleavage of Beclin1 creates destructive feedback, amplifying mitochondrial apoptosis. The central convergence on caspase-3 highlights apoptosis as the final common pathway for diverse pathological insults.

Mitochondria, the primary suppliers of ATP, demonstrate significant morphological and functional plasticity in neurons to meet metabolic demands and play a central role in apoptosis (Green and Reed, 1998). Mutations in key PD genes, including DJ-1, *α*-syn, Parkin, PINK1, and LRRK2, are highly associated with mitochondrial dysfunction (Haque et al., 2022). These mutations, in turn, lead to damage to various aspects of brain mitochondria in PD, including inhibition of mitochondrial biosynthesis and autophagy, imbalance in mitochondrial dynamics (specifically fusion versus fission), and underexpression of the mitochondrial import machinery (MIM), which consists of the translocase of the outer membrane (TOM) and the translocase of the inner membrane (TIM), among others (Selvaraj and Piramanayagam, 2019; Ryan et al., 2015; Goyani et al., 2024). Abnormalities in any of these can activate the mitochondrial apoptotic pathway typical of DA neurons in the midbrain in PD (Wright, 2022; Henrich et al., 2023). Typically, upon receiving apoptotic signals, the mitochondrial membrane undergoes changes including increased mitochondrial outer membrane permeabilization (MOMP) and a decrease in mitochondrial membrane potential (Δψm) (Garrido et al., 2006; Tait and Green, 2010). This results in the release of a substantial amount of Cyt C into the cytoplasm, along with Apaf-1 (Garrido et al., 2006; Tait and Green, 2010). Together, they form an apoptotic complex that subsequently recruits pro-Caspase-9 to form apoptotic vesicles (Green, 2022b). This initiates a caspase cascade that leads to apoptosis. Moreover, the release of additional apoptogenic factors such as the second mitochondria-derived activator of caspases (Smac), apoptosis-inducing factor (AIF), and endonuclease G (Endo G), in conjunction with Cyt C, exacerbates the apoptosis of DA neurons in PD (Green, 2022b; Bock and Tait, 2020). These factors collectively contribute to the pronounced susceptibility of midbrain neurons to mitochondrial dysfunction and apoptotic processes, underscoring the critical need for targeted therapeutic strategies to mitigate mitochondrial damage and restore neuronal health in PD (Figure 1).

### 3.2 Neuroinflammation and apoptosis

Neuroinflammation is a pivotal process in PD that involves the activation of glial cells in response to various stimuli (Tansey et al., 2022; Saadh et al., 2025; Kalia and Kuwar, 2026). This activation leads to the production of cytokines, chemokines, reactive oxygen and nitrogen species, second messengers, and the activation of the protein complement cascade (Vivekanantham et al., 2015). Together, this eventually contributes to decreased integrity of the BBB and subsequent uncontrolled neuronal death (Tansey et al., 2022). Currently, elevated levels of pro-inflammatory cytokines, including interleukin-1β (IL-1β), tumor necrosis factor-*α* (TNF-α), interferon-*γ* (IFN-γ), and interleukin-6 (IL-6) have been identified in the SN, chorionic nucleus, cerebrospinal fluid, and serum of both PD animal models and patients (Öberg et al., 2021; Teismann and Schulz, 2004). Reactive microglia and astrocytes were observed in postmortem brain samples from PD patients (Teismann and Schulz, 2004). Positron emission tomography also showed increased glial cell activation early in PD, which was inversely correlated with DA terminal density (Strafella et al., 2017).

Activation of glial cells leads to the formation of pro-inflammatory (M1-type) or anti-inflammatory (M2-type) responses (Kumar et al., 2025; Backman et al., 2025). Under anti-inflammatory conditions, microglia activate anti-inflammatory factors such as interleukin-10 (IL-10), and transforming growth factor-*β* (TGF-β) (Liu et al., 2019; Bartels et al., 2020). Similar to microglia, astrocytes secrete anti-inflammatory neurotrophic factors like brain-derived neurotrophic factor (BDNF), glial cell line-derived neurotrophic factor (GDNF), and mesencephalic astrocyte-derived neurotrophic factor (MANF), thus enhancing the viability and sustaining the survival of DA neurons (Wei and Shetty, 2021). However, in PD, a chronic neuroinflammatory state predominates, with a shift towards a pro-inflammatory glial phenotype (Wang B. et al., 2025; Krzisch et al., 2025; Lee et al., 2025). This shift results in the release of inflammatory mediators that contribute to the apoptosis and loss of DA neurons (Liu and Wei, 2025; Zhao et al., 2025). Numerous studies suggest that targeting neuroinflammation can reduce excessive apoptosis to alleviate the pathological changes and symptoms of PD (Wang W. et al., 2021; Cai et al., 2022). This strategy emphasizes the need to understand and manipulate the inflammatory processes within the PD brain to develop effective interventions (Figure 1).

### 3.3 Autophagy inhibition and apoptosis

Autophagy, a key degradation mechanism, is vital for maintaining protein homeostasis and recycling damaged organelles (Yamamoto et al., 2023; Aman et al., 2021). The autophagic process encompasses five distinct stages: initiation, extension, autophagosome maturation, fusion, and autolysosome formation (Wang W. et al., 2021). Triggered by both intra- and extracellular stresses, autophagy begins with the activation of the PI3K complex, including the key autophagy protein Beclin1, which facilitates the elongation of phagocytic vesicles (Pena-Martinez et al., 2022). During autophagosome maturation, LC3-II gets conjugated to the autophagic membrane, serving as a key marker for the formation of mature autophagosomes (Bekker et al., 2021). These autophagosomes subsequently fuse with lysosomes, creating autolysosomes that release lysosomal hydrolases to degrade cellular contents (Bekker et al., 2021). In the PD environment, the contribution of autophagy to the removal of accumulated *α*-syn, degenerating DA neurons and damaged mitochondria, as mentioned above, is indisputable (Lu et al., 2020; Irrcher and Park, 2009). Autophagy is divided into three types: macroautophagy, microautophagy, and chaperone-mediated autophagy (CMA), each defined by its own method for delivering cargo to lysosomes (Palmer et al., 2025; Gross et al., 2025). Both macroautophagy (Webb et al., 2003) and CMA (Vogiatzi et al., 2008) target the degradation of *α*-syn, particularly its pathogenic mutant forms like A30P and A53T (Cuervo et al., 2004). These mutations disrupt the normal binding function between chaperone proteins and CMA, leading to the accumulation of LBs in neurons (Cuervo et al., 2004). And, autophagy is inextricably linked to apoptosis (Marino et al., 2014). As previously observed, inhibition of autophagy is observed to increase the accumulation of α-syn aggregates, subsequently triggering the caspase cascade and inducing apoptotic neuronal death (Liu et al., 2017; Zhu et al., 2025; Kim Y. et al., 2025). Furthermore, therapeutic compounds such as Deferoxamine, Erinacine A, and Rutin are being explored for their potential to modulate autophagy-associated protein expression and signaling pathways, thus reducing the exacerbated neuronal apoptosis observed in PD animal models (Bekker et al., 2021; Zhu et al., 2024; Li X. et al., 2025; Wang H. et al., 2025; Niu et al., 2025; Shahid et al., 2025; Ayton et al., 2025) (Figure 1).

## 4 Interrelationship between exercise, apoptosis, and PD

### 4.1 Influence of exercise on PD pathophysiology

Given the limited clinical effectiveness of current pharmacological treatments for PD, researchers have been motivated to explore alternative therapeutic strategies for the prevention or management of this debilitating condition (Menozzi and Schapira, 2025; Zhang Y. et al., 2025; Winston et al., 2025; Szunyogh et al., 2025; Barker and Buttery, 2025; Jeon et al., 2025; Bronte-Stewart et al., 2025). Engaging in physical exercise has been recognized as a promising strategy for individuals with PD as many types of long-term endurance exercise therapy, including aerobic training, balance training, and resistance training, has shown efficacy in enhancing gait, balance, pain management, as well as addressing cognitive and psychological symptoms (both motor and non-motor), and thus quality of life (Wilson et al., 2025; Diaz-Galvan et al., 2026; van Duijvenboden et al., 2025; Luthra et al., 2025). Epidemiologic studies have shown that regular moderate-intensity aerobic exercise significantly reduces the risk of PD (Zhen et al., 2022; Ernst et al., 2023). Owing to the general lack of detailed guidelines in existing research literature concerning the optimal exercise protocol, Alberts conducted a comprehensive review of data from both animal and human studies (Alberts and Rosenfeldt, 2020). The review proposes that exercising three times per week for 30–40 min at moderate-to-high intensity, corresponds to 60–80% of heart rate reserve or 70–85% of maximum heart rate, could contribute to slowing the progression of PD (Alberts and Rosenfeldt, 2020). Mounting evidence now points to a two-way communication network between the gut and the brain, often termed the gut–brain axis, which has reshaped how we approach PD pathophysiology (Wang Q. et al., 2021; Travagli et al., 2020). Research further indicates that aging alters the composition of the gut microbiota, notably reducing populations of butyrate-producing bacteria (Tan et al., 2022; Gubert et al., 2020). This is significant because these microbes are thought to influence the aggregation of *α*-syn, a protein closely linked to PD (Tan et al., 2022; Gubert et al., 2020). Exercise, interestingly, has emerged as one possible way to modify the gut microbiome in neurodegenerative conditions, including PD (Morella et al., 2023). That said, clinical studies specifically examining how exercise affects the gut microbiota in PD patients remain scarce (Li T. et al., 2025; Baldauf et al., 2025; Peng et al., 2025), and the exact mechanisms behind its symptomatic benefits are still not well defined. Notably, in mouse models of PD, exercise has been shown to attenuate the loss of DA neurons (Kumar et al., 2025; Li et al., 2023; Ferreira et al., 2021; Johansson et al., 2022; Palasz et al., 2025; Xu J. et al., 2025; Kerdiles et al., 2025), implying that its protective effects may partly work by inhibiting certain forms of cell death.

### 4.2 Modulation of apoptotic pathways by exercise

Studies have revealed that these ameliorative effects may be realized through mechanisms such as increasing neuroplasticity, modulating neurotrophic factors and glutamate receptors, attenuating mitochondrial dysfunction (Li N. et al., 2025; Patki and Lau, 2011; Li B. et al., 2025), alleviating neuroinflammation (Wang B. et al., 2025; Kim et al., 2021; Zhao et al., 2025), and promoting cellular autophagy (Liu W. et al., 2020; Zhang X. et al., 2025; Liu M. et al., 2025). Abnormalities in any of these mechanisms, however, ultimately can lead to excessive neuronal apoptosis in PD. Exercise has been considered a potent modulator of apoptosis, and it is believed that the benefits of exercise in PD are likely to be achieved by inhibiting excessive neuronal apoptosis (Andreotti et al., 2020) (Table 2). Non-motor symptoms of PD can be ameliorated by exercise (Crowley et al., 2019). Ladder-climbing resistance training of ICR PD mice for 5 weeks markedly decreased the expression of the pro-apoptotic proteins cleaved Caspase-3 and Bax in the hippocampus, whereas the expression of the anti-apoptotic protein Bcl-2 was significantly increased (Kim et al., 2021). Furthermore, the buck-avoidance test indicated that exercise intervention prolonged the latency of PD mice in the platform. These data suggest that exercise reduces excessive neuronal apoptosis in the hippocampus as a way to improve spatial learning memory in PD mice (Kim et al., 2021). Also, motor symptoms can alleviated by exercise (van der Kolk and King, 2013). 6 weeks of treadmill running greatly enhanced the expression levels of the anti-apoptotic proteins Mcl-1 and Bcl-2 in C57BL/6 PD mice, and the hanging wire test indicated a significant improvement in neuromuscular function in the exercising PD mice (Jang et al., 2018b). As a result, exercise-induced reduction of excessive apoptosis in neuronal cells may represent a novel primary target for PD treatment (Lei et al., 2025; Chen W. et al., 2025; Lei et al., 2025). Nevertheless, the precise chemical process underlying the improvement remains unknown.

**Table 2:** Effects of exercise on apoptosis in patients or animal models of Parkinson’s disease.

Refs.	PD model	Tissue	Exercise type	Changes in apoptosis	Symptom changes
Liu W. et al. (2020)	6-OHDA treated male Sprague–Dawley rats (13 months old)	striatum	treadmill exercise, time increased from 15 min in the 1st week to 40 min in the 8th week, 5 days/week	Tunel-positive cells ↓ Apoptotic nuclei observed by the TEM ↓	the motor function was improved TH ↑ α-syn ↓
Jang et al. (2018a)	MPTP treated male C57BL/6 J mice (7 weeks old)		treadmill exercise, 60 min/day, 5 days/week for 8 weeks	cleaved Caspase-3, Bax ↓ Bcl-2 ↑	motor disfunction was restored TH ↑ α-syn ↓
Koo et al. (2017b)	MPTP treated male C57BL/6 J mice (7 weeks old)	substantia nigra	treadmill exercise, 60 min/day, 5 days/week for 8 weeks	Caspase-3 and cleavage ↓ Bcl-2 ↑	improved motor deficits and reduced TH, DAT ↑ α-syn ↓
Koo et al. (2017a)	MPTP treated male C57BL/6 J mice (7 weeks old)		treadmill exercise, 40–60 min/day, 5 days/week for 8 weeks	cleaved Caspase-3, Bax ↓ Bcl-2 ↑	improved motor deficits and reduced TH, DAT ↑ α-syn ↓
Cho et al. (2013)	6-OHDA treated female Sprague–Dawley rats	substantia nigra	treadmill exercise, 30 min/day for 14 consecutive days	Caspases-3 ↓	short-term memory was protected TH ↑
Rezaee et al. (2019)	6-OHDA treated male Wistar rats (2 months old)	striatum	treadmill exercise, 50 min/day, 5 days/week for 16 weeks	TFAM, p53 ↓	TH ↑
Kim et al. (2021)	MPTP treated male ICR mice (10 weeks old)	hippocampus	Resistance exercise, climb the ladder 5 days per week for 5 weeks	cleaved Caspase-3, Bax ↓ Bcl-2 ↑	cognitive function was improved
Lavin et al. (2020)	subjects with idiopathic PD	skeletal muscle	a combination of strength, power, endurance, balance, and functional training, 45 min/day, 3 days/week for 16 weeks	DNAJB4, SEC61A1, GBP2 (apoptosis related genes) ↓	SIPA1L2, LOXHD1, RASGRF, VIPR1 (genes linked to PD pathology) ↑

↑, activation or increase; ↓, inactivation or decrease. MPTP^+^, 1-methyl-4-phenyl-1,2,3,6-tetrahydropyridine^+^; 6-OHDA, 6-hydroxydopamine.

Beyond individual mechanistic pathways, several overarching limitations pervade the literature on exercise and apoptosis in PD. First, publication bias likely inflates the apparent consistency of findings. Studies reporting null or negative effects are rarely published, creating an illusion of uniform exercise benefits that may not reflect reality. Second, the predominance of MPTP-based models (8 of 8 animal studies in Table 2) raises concerns about model specificity. MPTP produces acute, severe dopaminergic toxicity that differs fundamentally from the chronic, progressive neurodegeneration in human PD. Whether exercise exerts comparable anti-apoptotic effects in more physiologically relevant models (for example, *α*-syn overexpression, aging-accelerated models) remains unknown. Third, the dose–response relationship between exercise and apoptosis inhibition has received minimal attention. Most studies employ a single exercise protocol, precluding determination of whether effects are threshold-dependent or follow a graded response. The one study that examined exercise intensity suggested that higher intensities produced greater benefits than moderate intensities, but systematic dose–response studies remain lacking (Jang et al., 2018b). Fourth, the durability of exercise effects beyond the intervention period is unexplored. Whether anti-apoptotic benefits persist after exercise cessation—a critical question for clinical application—has not been addressed in any study. Finally, the field lacks standardization in outcome measurement. Apoptosis is assessed through diverse methods (TUNEL staining, cleaved caspase-3 immunohistochemistry, Bax/Bcl-2 ratios, electron microscopy) with varying specificity and sensitivity. Direct comparisons between studies are complicated by this methodological heterogeneity, and the field would benefit from consensus recommendations for core outcome measures.

## 5 Molecular pathways: exercise-mediated regulation of apoptosis in PD

**Figure 2:**
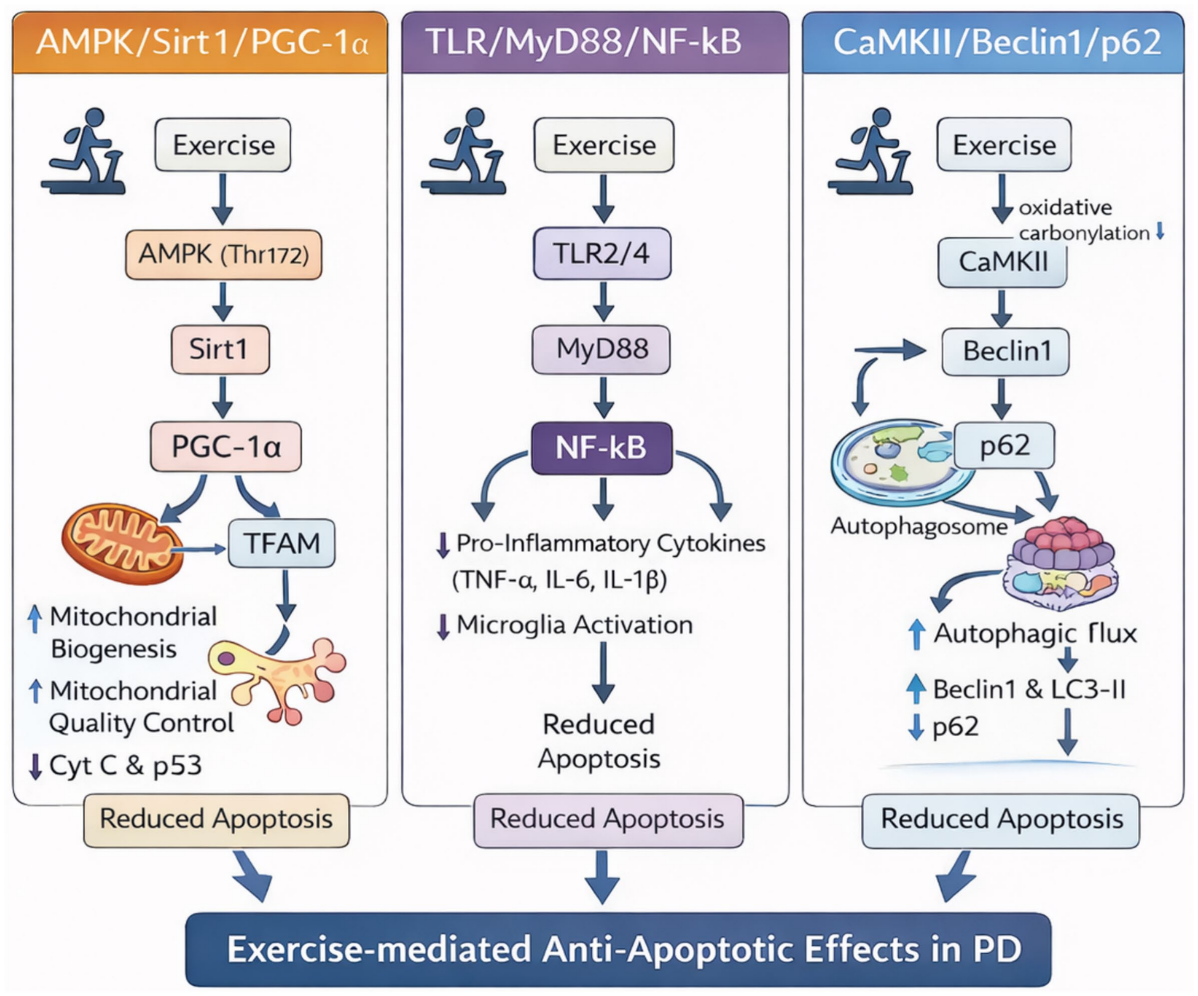
Flowchart of the experiment and alterations in the behavior and pathology of the mice. Exercise simultaneously engages three interconnected signaling pathways that converge to suppress neuronal apoptosis. AMPK/Sirt1/PGC-1α pathway (mitochondrial function): Exercise-induced energy stress activates AMPK, increasing Sirt1 activity and PGC-1α deacetylation. PGC-1α promotes mitochondrial biogenesis via TFAM, reducing cytochrome *c* release and p53-mediated apoptosis. TLR/MyD88/NF-κB pathway (neuroinflammation): Exercise suppresses TLR2/4 activation on microglia, inhibiting MyD88 recruitment and NF-κB nuclear translocation. This reduces pro-inflammatory cytokine production (TNF-α, IL-1β, IL-6) and subsequent death receptor activation. CaMKII/Beclin1/p62 pathway (autophagy): Exercise modulates CaMKII activity, promoting Beclin1-mediated autophagosome formation (LC3-II ↑) and autophagic flux (p62 ↓), enhancing clearance of damaged mitochondria and α-synuclein aggregates. Pathway crosstalk: AMPK directly phosphorylates ULK1 to initiate autophagy; Sirt1 deacetylates NF-κB subunits, dampening inflammation; inflammatory cytokines impair autophagic flux. Exercise creates a coordinated neuroprotective state by simultaneously engaging these pathways. Note: AMPK, AMP-activated protein kinase; Sirt1, sirtuin 1; PGC-1α, peroxisome proliferator-activated receptor *γ* coactivator-1α; TFAM, mitochondrial transcription factor A; TLR, Toll-like receptor; MyD88, myeloid differentiation factor 88; NF-κB, nuclear factor-κB; TNF-α, tumor necrosis factor-α; IL, interleukin; CaMKII, calcium/calmodulin-dependent protein kinase II; LC3-II, microtubule-associated protein 1 light chain 3II.

The anti-apoptotic effects of exercise in PD are mediated through three interconnected cellular processes: mitochondrial quality control, neuroinflammation, and autophagy. These processes do not operate in isolation but form an integrated network (Figure 2). Mitochondrial dysfunction generates reactive oxygen species that activate inflammatory pathways; neuroinflammation in turn impairs autophagic clearance; and defective autophagy fails to remove damaged mitochondria, perpetuating a vicious cycle of cellular stress and apoptotic vulnerability.

### 5.1 Exercise-AMPK/Sirt1/PGC-1α-mitochondrial dysfunction and apoptosis

Exercise has been shown to improve mitochondrial function by encouraging mitochondrial biogenesis (Chang et al., 2025; Tu, 2025), enhancing mitochondrial quality control via mechanisms such as mitophagy (Liu C. et al., 2025; Li Y. L. et al., 2025; Pradeepkiran et al., 2025), and increasing mitochondrial antioxidant capacities (Zhu et al., 2025; Trewin et al., 2018). These adaptations reduce oxidative stress and augment energy production in neurons, thereby offering protection against apoptosis (Fernandez-Checa et al., 2010). Specifically, exercise-mediated mitigation of excessive neuronal apoptosis in PD is partly due to the restoration of mitochondrial homeostasis, with mitochondrial biogenesis playing a critical role (Moradi Vastegani et al., 2023). The transcriptional coactivator peroxisome proliferator-activated receptor *γ* coactivator-1α (PGC-1α), pivotal in enhancing mitochondrial functions, orchestrates the oxidative defenses and governs the expression of mitochondrial biogenesis-related factors, including mitochondrial transcription factor A (TFAM) (Patki and Lau, 2011). Research including 18 weeks of treadmill exercise has shown significant reductions in the expression of apoptosis mediators Cyt C and p53 in the CS mitochondria of C57BL/6 PD mice, alongside increased levels of TFAM and PGC-1α (Patki and Lau, 2011). Further, silent information regulator 1 (Sirt1) enhances mitochondrial quality control by regulating PGC-1α transcription (Patki and Lau, 2011). Studies by Koo and Cho (2017) reveal that 8 weeks of treadmill running led to reduced Cyt C and p53 levels in striatal neurons of C57BL/6 PD mice, likely through the deacetylation effects of Sirt1, enhancing TFAM and PGC-1α activities. Moreover, a study by Rezaee et al. showed that 16 weeks of treadmill running significantly upregulated the expression of Sirt1, PGC-1α, and TFAM at both the gene and protein levels in the striatum of Wistar rats with PD (Rezaee et al., 2019). This regimen also reduced p53 expression and enhanced AMPKα phosphorylation at Thr 172 (Rezaee et al., 2019), indicating a positive role in PD. These findings show that exercise protects PD DA neurons against excessive apoptosis through the AMPK/Sirt1/PGC-1α signaling pathway, which targets mitochondrial biogenesis (Figure 2) Additionally, exercise has been shown to modulate the phosphorylation of mitochondrial dynamin-related protein 1 (Drp1) at Ser 637 (Jang et al., 2018b), as well as the expression of fusion-associated optic atrophy 1 (Opa1), mitofusin 2 (Mfn2) (Jang et al., 2018b), and core components of the TOM complex proteins TOM-20, TOM-40, and TIM-23 (Koo et al., 2017a), thereby contributing to the alleviation of excessive neuronal apoptosis in PD. However, despite these promising findings, the field still lacks extensive literature. Given the various triggers of mitochondrial dysfunction in PD, more experimental studies are required to delineate the particular pathways by which exercise ameliorates apoptosis by enhancing mitochondrial integrity in PD neurons.

### 5.2 Exercise-TLR/MyD88/NF-κB-neuroinflammation and apoptosis

Exercise has been demonstrated to have anti-inflammatory properties that may modulate neuroinflammation in the brain, potentially creating a neuroprotective environment conducive to neuronal survival (Zhao, 2024). This effect can be especially beneficial in conditions like PD, where inflammation plays an important part in neuronal injury (Zhang et al., 2023). By rebalancing pro-inflammatory and anti-inflammatory cytokines, exercise may contribute to slowing the progression of PD. Specifically, exercise has been shown to have favorable effects on brain regions other than the midbrain, which is often associated with the most severe PD lesions (de Laat et al., 2024). For example, 4 weeks of treadmill running has been reported to reduce excessive apoptosis of Purkinje cells in the cerebellar vermis of Sprague–Dawley PD rats (Lee et al., 2018). This reduction is linked to the inhibition of activated astrocytes and microglia, thereby improving motor balance and coordination.

At the molecular level, Nuclear factor-κB (NF-κB), a key player in the inflammatory and immune responses, also regulates cellular stress and apoptosis (Baska and Norbury, 2022). The accumulation of oligomerized *α*-syn in neurons, a hallmark of PD, is associated with NF-κB hyperactivation, which in turn triggers brain inflammation (Zubelzu et al., 2022). In experimental models, 5 weeks of stair-climbing resistance exercise was shown to inhibit NF-κB expression and the phosphorylation of its inhibitor (IκB-α) in hippocampal neurons. Concurrently, levels of inflammatory cytokines (TNF-α, IL-6, IL-1β) were significantly reduced, accompanied by normalized expression of key apoptosis-related proteins such as cleaved Caspase-3, Bax, and Bcl-2 (Kim et al., 2021). Furthermore, this exercise regimen helped moderate disrupted short-term memory in PD ICR mice, suggesting that exercise mitigates PD pathology by inhibiting NF-κB-mediated neuroinflammation and apoptosis (Kim et al., 2021). Additionally, Toll-like receptor 2/4 (TLR2/4), highly expressed on microglia membranes and up-regulated in PD, promotes the activation of myeloid differentiation factor 88 (MyD88) and downstream NF-κB signaling (Hughes et al., 2018). In C57BL/6 PD mice, 8 weeks of treadmill running significantly reduced the expression of TNF-*α*, IL-1β, phosphorylated IκB-α, and NF-κB in the CS, along with a marked decrease in TUNEL-positive apoptotic cells (Jang et al., 2017). Exercise also inhibited the activation of TLR2, MyD88, and reduced the pathology and symptoms in PD mice (Jang et al., 2017). This finding aligns with other experimental studies, including those by researchers like Koo et al. (2017b) and Wang W. et al. (2021), who noted co-localization of microglia with the inflammatory vesicle marker NLRP3 in the SN of PD mice and reported that treadmill running effectively inhibited microglial activation and NLRP3 expression (Wang W. et al., 2021). Collectively, these results demonstrate that exercise mitigates excessive apoptosis in PD DA neurons by suppressing neuroinflammation, largely through downregulating the TLR/MyD88/NF-κB pathway and subsequent inflammasome activation. Further research is needed to completely understand the processes by which exercise contributes to neuroprotection in PD, which could lead to effective therapeutic interventions (Figure 2).

### 5.3 Exercise-CaMKII/Beclin1/p62-autophagy inhibition and apoptosis

Failure of autophagy, a mechanism that normally clears toxic protein aggregates and damaged mitochondria, has been implicated in the pathogenesis of PD, as its dysregulation can promote neuronal apoptosis (Bekker et al., 2021). Exercise may serve as a therapeutic intervention for neurodegenerative conditions by positively modulating autophagic flux in the brain (Andreotti et al., 2020). Beclin1, the pioneering mammalian autophagy gene identified, plays a central role in the autophagy process (Prerna and Dubey, 2022). Its pro-autophagic activity is disrupted by the apoptotic protein caspase, which cleaves Beclin1. The resulting C-terminal fragment of Beclin1 thus takes on a new function, thereby amplifying mitochondria-mediated apoptosis (Djavaheri-Mergny et al., 2010). The Beclin1 downstream protein p62 delivers polyubiquitinated cargo to the autophagosome for lysosomal degradation, and thus reduced levels of p62 are thought to restore autophagic function (Koo and Cho, 2017). In C57BL/6 PD mice, 8 weeks of treadmill running significantly increased the protein levels of Beclin1 and LC3-II (a marker of autophagosome formation) in the SN and CS, while concurrently decreasing p62 expression (Koo and Cho, 2017). Concurrently, exercise also significantly lowered levels of the apoptotic proteins Cyt C and cleaved Caspase-3, indicating its neuroprotective effect (Koo and Cho, 2017). Jang et al. (2018a) further elucidated through correlation analysis that the neuroprotective effects of treadmill exercise are closely associated with enhanced autophagy flux. Specifically, the 8-week treadmill training regimen significantly upregulated the expression of BCL2 interacting protein 3 (BNIP3), a key mitochondrial autophagy protein—a key mitochondrial autophagy protein, alongside Beclin1 and LC3-II, both associated with pan-autophagy (Jang et al., 2018a). Concurrently, the expression levels of p62, an autophagy substrate, correspondingly decreased, collectively confirming the effective activation of the autophagy flux (Jang et al., 2018a). Moreover, post-translational modifications of Beclin1, such as phosphorylation, ubiquitination, and acetylation, influence its complex formation and consequently autophagic activity (Li et al., 2017). Calcium/calmodulin-dependent protein kinase II (CaMKII), a serine/threonine protein kinase, has been shown to modulate autophagy by binding directly to Beclin1 in neurons (Li et al., 2017). Further evidence by Liu and colleagues shows that 8 weeks of treadmill running significantly reduces TUNEL-positive apoptotic cells in the cortex of Sprague–Dawley PD rats (Liu W. et al., 2020). This reduction is associated with increased expression of Beclin1 and LC3-II, and attenuated carbonylation modification of CaMKIIα, suggesting a direct link between exercise-induced modifications in CaMKII activity and enhanced autophagy (Liu W. et al., 2020). In summarize, the CaMKII/Beclin1/p62 pathway is important in regulating the effects of exercise on autophagy increase and apoptosis inhibition in PD DA neurons. These findings highlight the therapeutic potential of tailored physical activity in PD by modulating autophagic pathways and improving neuronal survival (Figure 2).

The relationship between exercise-enhanced autophagy and reduced apoptosis appears intuitively appealing, but causal evidence remains limited. Current studies demonstrate correlational associations, exercise increases Beclin1 and LC3-II while decreasing p62 and apoptotic markers, but do not establish that enhanced autophagy directly mediates apoptosis suppression. The critical experiment, using autophagy inhibitors or genetic knockout models to determine whether exercise benefits persist when autophagy is blocked, has not been performed. Without such loss-of-function studies, the proposed causal chain remains speculative.

The three pathways described above do not operate in isolation but exhibit extensive bidirectional crosstalk that amplifies exercise benefits. AMPK activation, the central node in exercise responses, not only promotes mitochondrial biogenesis via PGC-1α but also directly phosphorylates Unc-51 like autophagy activating kinase 1 (ULK1) to initiate autophagy (Kim et al., 2011). Sirt1, activated downstream of AMPK, deacetylates both PGC-1α and autophagy components (for example, autophagy related genes 5 and 7, ATG5, ATG7), coordinating mitochondrial and autophagic adaptations (Lee et al., 2008). Conversely, NF-κB-mediated inflammation suppresses autophagy by sequestering essential autophagy proteins such as Beclin1 (Xiao, 2007), while dysfunctional mitochondria release damage-associated molecular patterns that perpetuate TLR/NF-κB activation (Gao et al., 2024). This interdependence creates a vicious cycle in PD pathology: mitochondrial dysfunction triggers inflammation, inflammation impairs autophagy, and failed autophagy fails to remove damaged mitochondria. Exercise, by simultaneously engaging AMPK/Sirt1 signaling while suppressing NF-κB, may break this cycle and create a coordinated neuroprotective response rather than isolated effects on individual pathways.

## 6 Translational considerations: from bench to bedside

The preclinical evidence summarized above provides compelling mechanistic insights into how exercise may suppress neuronal apoptosis in PD. However, translating these findings to clinical practice requires critical examination of the substantial gap between animal models and human disease.

### 6.1 Limitations of current animal models

The predominant use of toxin-based models in exercise research (Tables 1, 2) presents fundamental translational challenges. These models induce acute, severe dopaminergic degeneration over days to weeks, whereas human PD progresses over decades with gradual accumulation of *α*-syn pathology. The cellular stress responses triggered by acute toxin exposure may differ qualitatively from those in chronic neurodegeneration, potentially altering exercise effects. For instance, MPTP produces rapid mitochondrial complex I inhibition and oxidative burst, eliciting apoptotic cascades within 24–72 hours (Jackson-Lewis and Przedborski, 2007; Liberatore et al., 1999). In contrast, human DA neurons face sustained, low-level stress from α-syn oligomers, impaired proteostasis, and age-related mitochondrial dysfunction (Kim et al., 2022). Whether exercise can modify this chronic, low-grade apoptotic process remains unclear.

Furthermore, animal studies predominantly use young adult rodents (7–13 weeks old, equivalent to human adolescence/early adulthood) (Jackson-Lewis and Przedborski, 2007; Zhang et al., 2022), despite PD being predominantly a disease of aging. Aged animals exhibit diminished neuroplasticity, reduced autophagic capacity, and enhanced inflammatory responses (Zhuang et al., 2013)—factors that likely modify exercise responsiveness (Chen X. et al., 2025). The single study using older rats (13 months, equivalent to middle-aged humans) reported attenuated exercise effects compared to younger animals in historical controls (Liu W. et al., 2020), suggesting age may blunt anti-apoptotic benefits. Systematic comparisons across age groups are urgently needed.

The genetic homogeneity of inbred rodent strains (C57BL/6, Sprague–Dawley, Wistar) also fails to capture the genetic diversity of human PD patients, including those with risk variants in SNCA, LRRK2, GBA, and other genes (Chen et al., 2020). Exercise effects may differ substantially between individuals with different genetic backgrounds, yet this heterogeneity remains unexplored in preclinical models.

### 6.2 Methodological discrepancies between animal and human studies

Exercise protocols in animal research bear limited resemblance to human interventions. Rodent studies employ forced treadmill running at fixed speeds and durations (typically 40–60 min/day, 5 days/week) (Jang et al., 2018a; Koo et al., 2017b; Koo et al., 2017a), representing a standardized but artificial form of exercise. Human patients engage in voluntary, varied-intensity activities (walking, cycling, resistance training, balance exercises) that differ in metabolic demand, muscle recruitment patterns, and neuroendocrine responses. Whether treadmill running optimally models the human exercise experience, or whether certain exercise modalities translate more effectively, remains unknown.

The outcome measures also differ fundamentally. Animal studies directly assess neuronal apoptosis in brain tissue—a gold standard impossible to achieve in living humans. Human studies rely on indirect measures: functional outcomes (motor performance, cognition), structural neuroimaging (dopamine transporter PET, volumetric MRI), or peripheral biomarkers (blood-based proteins, gene expression in accessible tissues like skeletal muscle) (Cropley et al., 2006; Kaasinen and Rinne, 2002). The relationship between these indirect measures and neuronal apoptosis is inferential at best. Lavin et al. reported downregulation of apoptosis-related genes in skeletal muscle of exercising PD patients, but whether this reflects similar changes in brain DA neurons is unknown (Lavin et al., 2020). Emerging biomarkers such as neuron-derived extracellular vesicles carrying caspase-cleaved products offer promise but require validation (Garrido et al., 2006).

### 6.3 Strategies to bridge the translational gap

Addressing the translational disconnect between preclinical findings and clinical application requires a coordinated, multi-level research strategy.

At the preclinical level, future studies must prioritize more translationally relevant models. While toxin-based models (MPTP, 6-OHDA) have provided foundational insights, they fail to recapitulate the chronic, progressive nature of human PD. Incorporating aged animals (≥18 months) is essential to determine whether exercise benefits persist in organisms with age-related decline. Genetic models (*α*-syn transgenic, LRRK2 mutation carriers) should complement toxin studies to reflect human disease heterogeneity. Both sexes must be included given emerging evidence of sexual dimorphism in PD pathophysiology and exercise responses.

Experimental design must also evolve. Dose–response studies are needed to establish minimum effective dose and optimal intensity, rather than relying on single-protocol comparisons. Longitudinal designs with multiple timepoints would elucidate temporal dynamics of apoptosis regulation. Exercise cessation studies are critical to determine whether benefits persist after intervention withdrawal, a question with direct clinical implications.

At the clinical level, advancing translation requires validated mechanistic biomarkers. Neuron-derived extracellular vesicles (NDEVs) isolated from peripheral blood offer a promising avenue, as these carry caspase products and microRNAs that may report on neuronal health (Eitan et al., 2023; Saeedi et al., 2021). Until such tools are validated, human studies should integrate multi-modal assessments combining neuroimaging (dopamine transporter PET) with blood-based biomarkers and functional outcomes.

In summary, bridging the translational gap requires an integrated framework that embraces biological complexity, leverages emerging technologies, and maintains continuous dialogue between preclinical and clinical investigation.

## 7 Perspectives

This review systematically summarizes the molecular mechanisms by which exercise suppresses excessive neuronal apoptosis in PD through regulating mitochondrial function (AMPK/Sirt1/PGC-1α), neuroinflammation (TLR/MyD88/NF-κB), and autophagy (CaMKII/Beclin1/p62). These pathways do not operate in isolation but form an integrated network, with AMPK serving as a central hub coordinating mitochondrial biogenesis, autophagic flux, and anti-inflammatory responses. Given the ameliorative effects of exercise on PD pathology and symptoms, coupled with the critical role of these processes in mediating the anti-apoptotic effects of exercise, apoptosis itself is highly likely to represent a core pivotal mechanism in exercise-based prevention and treatment of PD (Chen X. et al., 2025; Li N. et al., 2025; Palasz et al., 2025). Deepening our understanding of these molecular interactions will aid in exploring how different exercise types, intensities, and durations can optimize their neuroprotective effects on PD.

From an exercise physiology perspective, existing research has primarily focused on the effects of long-term treadmill training on PD animal models (Chen X. et al., 2025; Fukuchi et al., 2026; Nhu et al., 2021; Handlery et al., 2021), while the differential modulatory effects of different exercise modalities on apoptosis remain unknown. Aside from one study on resistance training, the impacts of other exercise forms—such as interval training and balance training—have yet to be clarified. Although this review does not systematically categorize exercise types, it emphasizes the necessity for in-depth research in this area. This will facilitate the future development of personalized exercise programs tailored to individual circumstances and preferences, thereby enhancing intervention adherence and efficacy.

From a translational perspective, several critical questions remain: (1) whether exercise intensities optimized in rodents (40–60 min/day, 5 days/week) are directly applicable to human patients, (2) whether anti-apoptotic effects observed in young adult animals persist in aged organisms, and (3) how genetic heterogeneity (SNCA, LRRK2, GBA variants) modulates individual responses to exercise. Addressing these questions will require carefully designed clinical trials incorporating mechanistic biomarkers (neuron-derived extracellular vesicles) and stratified patient populations.
